# Artificial Intelligence and Computer Aided Diagnosis in Chronic Low Back Pain: A Systematic Review

**DOI:** 10.3390/ijerph19105971

**Published:** 2022-05-14

**Authors:** Federico D’Antoni, Fabrizio Russo, Luca Ambrosio, Luca Bacco, Luca Vollero, Gianluca Vadalà, Mario Merone, Rocco Papalia, Vincenzo Denaro

**Affiliations:** 1 Unit of Computer Systems and Bioinformatics, Università Campus Bio-Medico di Roma, Via Alvaro Del Portillo, 21, 00128 Rome, Italy; f.dantoni@unicampus.it (F.D.); l.bacco@unicampus.it (L.B.); l.vollero@unicampus.it (L.V.); 2 Department of Orthopaedic Surgery, Università Campus Bio-Medico di Roma, Via Alvaro Del Portillo, 200, 00128 Rome, Italy; l.ambrosio@unicampus.it (L.A.); g.vadala@policlinicocampus.it (G.V.); r.papalia@policlinicocampus.it (R.P.); denaro@policlinicocampus.it (V.D.); 3 ItaliaNLP Lab, Istituto di Linguistica Computazionale “Antonio Zampolli”, National Research Council, Via Giuseppe Moruzzi, 1, 56124 Pisa, Italy; 4 Webmonks S.r.l., Via del Triopio, 5, 00178 Rome, Italy

**Keywords:** low back pain, orthopaedics, artificial intelligence, deep learning, decision support systems, computer aided diagnosis

## Abstract

Low Back Pain (LBP) is currently the first cause of disability in the world, with a significant socioeconomic burden. Diagnosis and treatment of LBP often involve a multidisciplinary, individualized approach consisting of several outcome measures and imaging data along with emerging technologies. The increased amount of data generated in this process has led to the development of methods related to artificial intelligence (AI), and to computer-aided diagnosis (CAD) in particular, which aim to assist and improve the diagnosis and treatment of LBP. In this manuscript, we have systematically reviewed the available literature on the use of CAD in the diagnosis and treatment of chronic LBP. A systematic research of PubMed, Scopus, and Web of Science electronic databases was performed. The search strategy was set as the combinations of the following keywords: “Artificial Intelligence”, “Machine Learning”, “Deep Learning”, “Neural Network”, “Computer Aided Diagnosis”, “Low Back Pain”, “Lumbar”, “Intervertebral Disc Degeneration”, “Spine Surgery”, etc. The search returned a total of 1536 articles. After duplication removal and evaluation of the abstracts, 1386 were excluded, whereas 93 papers were excluded after full-text examination, taking the number of eligible articles to 57. The main applications of CAD in LBP included classification and regression. Classification is used to identify or categorize a disease, whereas regression is used to produce a numerical output as a quantitative evaluation of some measure. The best performing systems were developed to diagnose degenerative changes of the spine from imaging data, with average accuracy rates >80%. However, notable outcomes were also reported for CAD tools executing different tasks including analysis of clinical, biomechanical, electrophysiological, and functional imaging data. Further studies are needed to better define the role of CAD in LBP care.

## 1. Introduction

In the last few decades, Artificial Intelligence (AI) has been revolutionizing the healthcare industry thanks to innovative computational tools able to support and even substitute human intelligence in some specific tasks [1]. To date, AI is being applied to almost any aspect of daily life, thanks to its capacity to handle the unprecedented amount of information recorded every nanosecond by computer systems, e.g., vocal assistants, car security devices, and smart home detectors. Due to the huge quantity of data and the ever-increasing use of digital processing in clinical practice, the employment of AI in medical research has been increasingly investigated in several studies [2]. Indeed, AI-based systems have been shown to perform automatic segmentation and data extraction from radiological datasets [3] as well as to support diagnosis, treatment, and outcome evaluation in different fields, including spine surgery [2].

The use of AI in spine surgery has been exploited for different tasks, including the segmentation of spinal structures [4], identification of degenerated discs [5], detection of vertebral fractures [6], classification of scoliotic curves [7], and several more. In a previous systematic review on the application of Computer Vision to the management of low back pain (LBP), we have demonstrated that AI systems achieved Sørensen–Dice scores >90% with regard to segmentation of vertebrae, intervertebral discs (IVDs), spinal canal, and lumbar muscles, whereas studies focusing on structure localization and identification demonstrated an accuracy >80% [8].

LBP is primarily caused by intervertebral disc degeneration, representing the main cause of disability in the world, with a huge impact on patients’ quality of life as well as on socioeconomic and working conditions [9]. Diagnosing and treating LBP often requires a multidisciplinary approach involving the acquisition of radiological images, patient-reported outcome (PROMs) evaluation questionnaires, and angular and linear measurements. Therefore, the ultimate decision is often guided by the elaboration of several data using an algorithmic approach [10,11]. Computer-aided diagnosis (CAD) is a field of AI employing machine learning methods to specifically analyze both imaging and non-imaging data in order to classify patients’ conditions and to support clinicians in the formulation of a correct diagnosis [12]. While having been firstly adopted for the diagnosis of breast cancer [12], CAD systems are now utilized in several fields, including the detection of osteoporosis [13], individuation of missed polyps during colonoscopy [14] and many others. Applications of CAD to LBP are numerous and involve several data sources (e.g., magnetic resonance imaging—MRI—and computed tomography—CT—datasets, clinical notes, surface sensor and electrophysiological measurements), as well as numerous ancillary AI tasks (e.g., segmentation, classification, regression).

The diagnosis of disc abnormalities can be easily performed by an experienced professional, even if affected by notable variability among experts (Alomari et al. [15] report that “there is over 50% inter- and intra-observer variability in the MRI interpretation that urges the need for standardized mechanisms in MRI interpretation”). This aspect can be automatized in AI systems specifically focusing on Computer Vision, with encouraging results from preliminary studies. For example, Won et al. [16] reported: “Spinal stenosis Grading agreement between the experts was 77.5% and 75.0% in terms of accuracy and F1 scores”. The main advantage of CAD systems is to carry out multiple tasks on large datasets resulting in a definite outcome with a high degree of accuracy when compared to the human counterpart. However, the real added value of AI in CAD systems is to combine different pieces of information (demographics, patient-reported outcome measures, clinic notes, radiological data, etc.) in order to better predict a specific diagnosis and improve patient outcomes. All of these aspects have been recently reviewed by Mallow et al. [17]. Briefly, although clinicians achieve high accuracy scores in some easy tasks such as detecting disc bulging, AI models achieve very similar results while reducing the diagnosis time, as well as excluding inter- and intra-observer variability. In addition, the diagnosis of some diseases is still challenging for medical practitioners, and can actually be aided and improved by AI.

In this review, we have systematically reviewed the available literature on the application of CAD systems to the management of LBP. The state of the art on the present technology and individual results of included studies will be thoroughly discussed, in order to describe the actual evidence and potential future applications of these ground-breaking tools.

## 2. Materials and Methods

In order to perform an exhaustive research of AI articles related to LBP, we performed literature research on PubMed, Scopus, and Web of Science. The search keywords utilized for both the medical and the AI part are reported in Table 1. At least one of the search keywords for the medical and the AI part had to be included in the title or in the abstract of the articles.

### 2.1. Inclusion and Exclusion Criteria

The aim of this study was to gather all the articles concerning the utilization of AI in the diagnosis of LBP and lumbar degenerative diseases. Straightforwardly, all the selected articles had to meet all the following inclusion criteria:

- Chronic LBP or lumbar degenerative diseases must have been between the main topics of the article. We included articles on the diagnosis of diseases related to chronic LBP, and treating at least one of the structures involved in LBP (i.e., vertebrae, discs, muscles, spinal canal);
- AI must have been used in the article. We included articles exploiting AI methods falling in the areas of computer vision, machine learning and neural networks (NNs), regardless of the type of data utilized (e.g., images, text data, clinical data);
- Aim of the study: all the articles included must have been focused on a CAD system;
- Subjects included in the study: all the articles must have been based on studies of human low back and related pathology, regardless of age or employment of the included individuals;
- Validation procedures: results must have been reported on a test set different from the training set;
- Language: all articles must have been written in English.

Conversely, articles that were excluded did not meet the inclusion criteria for one of the following reasons:

- A different medical problem was considered: we excluded articles which did not consider chronic LBP and its related anatomical structures and medical data. For example, we excluded studies that focused only on cervical or thoracic vertebrae, and studies focusing on acute LBP and osteoporosis;
- AI was not considered: we excluded studies that did not utilize AI-based techniques in the diagnosis or management of LBP;
- Diagnosis was not provided: we excluded studies using Computer-Vision-based methods that, although focusing on LBP related structures, limited to the segmentation or identification of lumbar structures;
- Animal studies: we excluded studies based on vertebral structures of animals, e.g., goats or mice;
- Results reproducibility: we excluded articles that did not use a K-fold cross-validation procedure or reported a clear division of the dataset between a training set and a test set.;
- Not in English: we excluded all the articles written in a language different from English.

**Figure 1:**
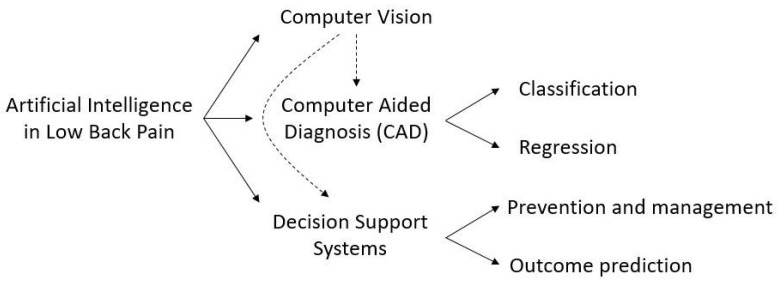
Partitioning of the studies concerning the application of AI in LBP, presented in [8].

In our previous study, we have defined three main categories in which the utilization of AI in LBP can be split, namely Computer Vision, CAD, and Decision Support Systems (DSS) (Figure 1). Computer Vision is the field of AI that deals with how computers can gain a high-level understanding from digital images or videos. With regard to LBP, its main applications concern feature extraction and image segmentation, which have been widely discussed in our previous systematic review [8].

CAD is a group of techniques which help medical practitioners identify a pathology or quantify the grade of a disease. It can be divided into two distinct tasks, namely classification and regression, in which machine or deep learning models are used to assign a predefined label or to generate a numeric output, respectively. In practice, classification is used to identify or categorize a disease, whereas regression is used to produce a numerical output as a quantitative evaluation of some measure [18].

DSS are systems that allow medical practitioners or patients to enhance the decision-making process in order to improve the outcome of subjects suffering from a specific disease. The goal of the vast majority of DSS is outcome prediction, i.e., the prediction of the improvement that a patient would experience after exposure to a defined therapy. By predicting the extent to which a patient would benefit from a specific treatment, DSS may provide the physician with practical tools to assess, for example, whether or not surgery may be preferable to conservative treatment. However, a DSS only provides a suggestion to the physician, who is responsible for the final decision on the treatment to be undertaken. Finally, DSS can be used for prevention, e.g., by providing the user with recommendations or correct practice for preventing the onset of a disease [19].

### 2.2. Evaluation Metrics

Among the articles included, different tasks resorted to different metrics to evaluate the performance of systems under investigation. However, considering the large amount of studies reported in this review, different metrics were also considered within the same task.

With regard to the Classification task, we reported the results in terms of Accuracy (Acc), where available. For brevity purposes, let us consider a binary Classification task, e.g., Positive vs. Negative. Given a test set composed of *N* samples, defining the True Positives TP as the number of Positive samples correctly classified, and the True Negatives TN as the number of Negative samples correctly classified, Accuracy is defined as:

(1) $$\mathrm{Acc}\%=\frac{TP+TN}{N}\times 100$$

Thus, greater values correspond to better performance. For each class, *Recall* and *Precision* can be computed as well. Defining the False Positives FP and False Negatives FN as the number of misclassified Positive/Negative samples, *Recall* and *Precision* are defined as:

(2) $$Recall=\frac{TP}{TP+FN}\;Precision=\frac{TP}{TP+FP}$$

in binary problems, *Recall* is also called True Positive Rate and corresponds to sensitivity, whereas the True Negative Rate is also called specificity. In the case of multi-class problems, accuracy is computed by considering the TP for each class, and *Recall* and *Precision* per class can be computed. Another widely used evaluation metric is the Area Under the Curve (AUC), which corresponds to the area under the Receiver Operating Characteristic (ROC) curve showing the performance of a classification model at all classification thresholds, which is plotted considering the True Positive Rate against the False Positive Rate. Its values range from 0 to 1 (the closer to 1, the better the performance).

With regard to the Regression task, the vast majority of the studies included in this review report the performance in terms of the Mean Absolute Error (*MAE*). Let us consider a sequence of original values x(t) and a sequence of predicted values $\tilde{x}\left(t\right)$. The MAE for a sequence of *N* timestamps is defined as:

(3) $$MAE=\sum_{t=1}^{N}\frac{|x\left(t\right)-\tilde{x}\left(t\right)|}{N},\;$$

Thus, the closer the value is to 0, the better the performance.

In some cases, percentage error values are used to evaluate performance, the meaning of which varies according to the investigated task.

### 2.3. Quality of Evidence

**Figure 2:**
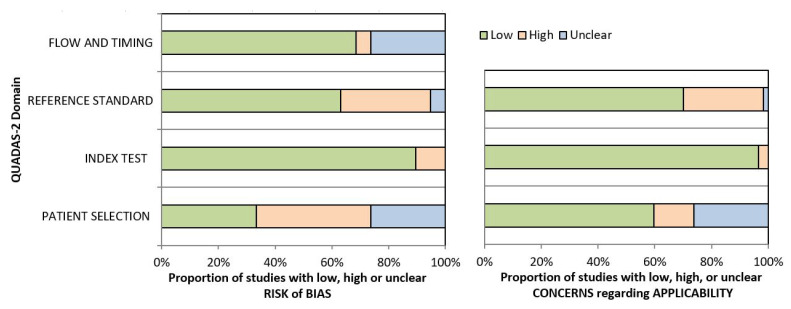
Summary of the methodological quality of included studies regarding the four domains assessing the risk of bias (**left**) and the three domains assessing applicability concerns (**right**) of the QUADAS-2 score. The portion of studies with a low risk of bias is highlighted in green, the portion with an unclear risk of bias is depicted in blue, and the portion with a high risk of bias is represented in orange.

The methodological quality of the included studies was assessed independently by two reviewers (L.A. and F.R.), and any disagreement was solved by the intervention of a third reviewer (G.V.). The risk of bias and applicability of included studies were evaluated by using customized assessment criteria based on the Quality Assessment of Diagnostic Accuracy Studies (QUADAS-2) [20]. This tool is based on four domains: patient selection, index test, reference standard, and flow and timing. Each domain is evaluated in terms of risk of bias, and the first three domains are also assessed in terms of concerns regarding applicability. Fifty studies were rated on a 3-point scale, reflecting concerns about risk of bias and applicability as low, unclear or high, as shown in Figure 2 (the details of the analysis are presented in Tables S1 and S2 in the Supplementary Materials).

## 3. Results

**Figure 3:**
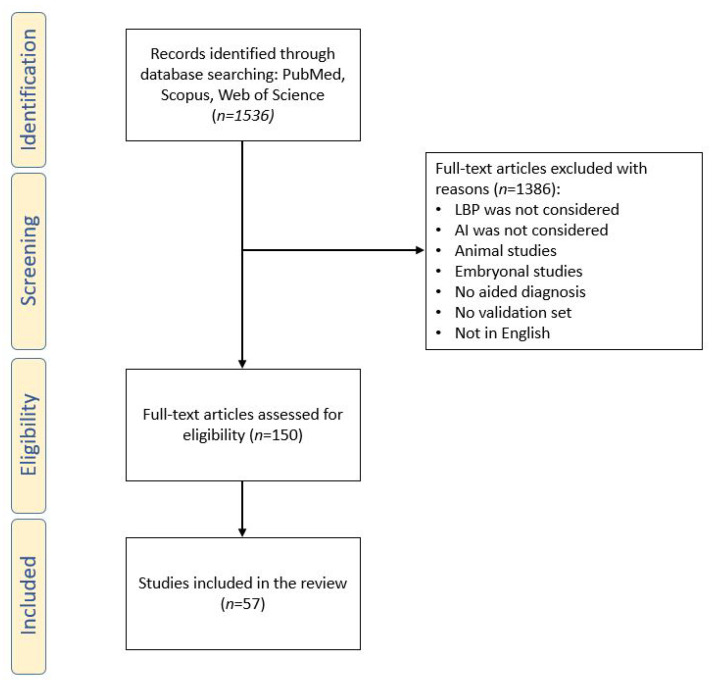
Preferred reporting items for systematic reviews and meta-analyses (PRISMA) flow diagram.

The search was performed on 5 November 2021, and resulted in 1536 articles. Nonetheless, after removing duplicates and following a first screening based on the article titles and abstracts, we reduced the number of eligible articles to 93, as many of them focused on a different topic. A second screening phase was performed after having read the full text of each article, which led the total amount of included articles to 57. We created a flow-chart diagram according to the PRISMA protocol that shows the selection process of the studies (Figure 3). The articles were screened by two independent reviewers and, in the case of discrepancies regarding the inclusion or exclusion of an article, they discussed together until consensus was reached. It is worth noting how the amount of published papers is increasing year by year, and that the number of articles published in 2020 is almost double compared to 2019. This may be due to two main reasons: first, the ever-increasing amount of clinical images and data available to researchers and, secondly, the improvement of computing capacity observed in recent years.

### 3.1. Computed Aided Diagnosis

Computer Aided Diagnosis (CAD) is a branch of AI that resorts to machine learning techniques to help physicians diagnose a disease or quantify its severity. Several studies resulted from the search utilized CAD systems, and they considered two main tasks, namely classification and regression. CAD systems can be based on clinical and physiological data and/or on clinical images, and, in the latter case, may be following a segmentation phase. In this systematic review, we found a total of 57 articles employing CAD systems, 45 of which were based on classification, and 12 were based on regression.

#### 3.1.1. Classification

Classification is a task that resorts to assigning an input sample to one of a finite number of predetermined classes, and can be based on machine learning models such as Support Vector Machines (SVM) and Decision Trees, or on deep learning models. In this review, we identified a total of 45 papers performing a classification task as a CAD, and their main features are reported in Table 2. Briefly, we included:

- 27 studies on clinical lumbar imaging, and in detail:
  − 20 studies on MRI;
  − 4 studies on X-ray images;
  − 3 studies on other typologies of medical images;
- 4 studies on brain MRI (1 in combination with other physiological data);
- 14 studies on physiological data, and in detail:
  − 8 studies using kinematic variables or sensor data;
  − 3 studies using clinical data and text notes;
  − 3 studies using electromyography (EMG) data.

Specifically, 18 out of the 45 papers focused on LBP diagnosis, 13 studies investigated disc degeneration, 4 studied spinal stenosis, and 3 approached spondylolisthesis, whereas the remaining studies focused on different conditions such as scoliosis, osteoarthritis, disc and bone diseases, and routine reporting. It is worth noting that 22 studies exploited NNs and deep learning, 22 exploited machine learning models, and 1 study exploited both approaches.

With regard to the studies focusing on the diagnosis of LBP, 4 articles utilized brain MRI to identify morphological factors predicting LBP, whereas 8 studies exploited other types of data such as EMG signals, kinematic variables or bio-mechanical measures; 3 papers were based on clinical data and text notes, and 3 studies aimed to diagnose LBP based on clinical images related to the lumbar region. All of the studies exploiting brain MRI chose a Support Vector Machine (SVM) as a classifier to discriminate between healthy and unhealthy subjects. In detail, Lee et al. [21] used brain MRI in combination with physiological parameters of 53 subjects to discriminate between healthy and LBP subjects, achieving an accuracy of 92.5%; Lamichhane et al. [22] searched for multimodal biomarkers of LBP on brain MRI images of 24 patients and 27 healthy control subjects with an accuracy of 78.7%; in addition, the same group [23] expanded the previous work by adding a Enet-subset feature selection, improving the SVM accuracy to 83.1%. Shen et al. [24] searched for alterations in brain functional connectivity due to chronic LBP, achieving 79.3% accuracy on brain MRI images of 90 patients.

Among the studies that aimed to diagnose LBP based on clinical data, Mathew et al. [25] used Inductive Learning in an early study to diagnose LBP based on clinical data from 200 subjects, achieving accuracy values ranging between 82 and 90%. Staartjes et al. [26] performed a Fuzzy-rule based classification based on Chi’s method to diagnose LBP based on clinical data from 262 subjects, with an accuracy of 96.2%. Parsaeian et al. [27] compared a Feedforward NN and Logistic Regression on clinical data from more than 34,000 subjects to diagnose LBP, achieving an equal AUC of 0.75.

**Table 2:** Summary Table of the works performing Classification. If more than one structure/task were investigated in a study, the correspondent results are reported in the same order in which the structures are presented in the “Structures involved”/“Target” column.

Author/Year	Data Type	# Patients	Structures Involved	Target	Results	Model
Lewandrowski, 2020 [28]	MRI	17,800 discs	Discs	Routine reporting	Acc = 85.2%	Tiramisu NN and CNN
Gao, 2020 [5]	MRI	500	Discs	Disc degeneration	Acc = 86%	CNNs:VGG-M, VGG-16, GoogLeNet, and ResNet-34
Ruiz-España, 2015 [29]	MRI	67	Discs	Disc degeneration	Acc > 90%	Gradient Vector Flow, several ML models
Oktay, 2014 [30]	MRI	102	Discs	Disc degeneration	Acc = 92.8%	SVM
Alomari, 2010 [31]	MRI	80	Discs	Disc degeneration	Acc = 91.3%	Probabilistic Gibbs model
Koh, 2012 [32]	MRI	70	Discs	Disc degeneration	Acc = 99%	Ensemble of ML models
Tsai, 2021 [33]	MRI	168	Discs	Disc degeneration	Acc = 81.1%	YOLOv3 CNN
Pan, 2021 [34]	MRI	500	Discs	Disc degeneration	Acc = 88.8%	Faster Region-based CNN
Beulah, 2021 [35]	MRI	93	Disc	Disc degeneration	Acc = 92.5%	Gabor features + SVM
Sundarsingh, 2019 [36]	MRI	63	Disc	Disc degeneration	Acc = 94.7%	Random Forest
Salehi, 2019 [37]	MRI	50	Discs	Disc degeneration	Acc = 97.9%	Active Contour + K-Nearest neighbors
Šušteršič, 2020 [38]	Force sensor data	33	Discs	Disc degeneration	Acc = 85%	Decision Tree
Rankovic, 2015 [39]	Force sensor data	38	Discs	Disc degeneration	Acc = 88.9%	Adaptive Network based Fuzzy Inference System
Oyedotun, 2016 [40]	Biomechanical measures	UCI MLR 310	Discs	Disc degeneration	Acc = 92.5 and 96.8%	Feedforward NN
Jamaludin, 2017 [41]	MRI	Genodisc 2009	Several structures	Disc and bone diseases	Acc = 71.5, 75.0, 95.2, 94.3, 86.3, 90.7%	CNN
Jamaludin, 2017 [42]	MRI	2009	Several structures	Disc and bone diseases	Acc = 70.1, 75.4, 95.4, 94.7, 87.5, 89.4%	CNN
Lehnen, 2021 [43]	MRI	146	Several structures	Disc and bone diseases	Acc = 87, 86, 76, 98, 91, 87.6%	U-net + image comparison
Han, 2018 [44]	MRI	200	Spinal canal	Spinal stenosis	Precision = 84.5%	CNN (DMML-Net)
Huber, 2009 [45]	MRI	82	Spinal canal	Spinal stenosis	Sensitivity = 94%, Specificity = 98%	Several ML algorithms
Hallinan, 2021 [46]	MRI	446	Spinal canal	Spinal stenosis	Acc = 96, 92 and 89%	CNN
Won, 2020 [16]	MRI	542	Spinal canal	Spinal stenosis	Acc = 83.0 or 77.9%	CNN
Veronezi, 2011 [47]	X-rays	206	Vertebrae	Osteoarthritis diagnosis	Acc = 62.9%	Feedforward NN
Adankon, 2012 [48]	3D image of the back surface	165	Vertebrae	Scoliosis diagnosis	Acc = 95%	Local Geometric Descriptors and SVM
Lin, 2007 [49]	X-rays	37	Vertebrae	Scoliosis diagnosis	Identification rate = 84%	Feedforward NN
Zhao, 2019 [50]	MRI	150	Vertebrae	Spondylolisthesis	Acc = 89.3%	Adversarial Recognition Network
Varcin, 2019 [51]	X-rays	286	Vertebrae	Spondylolisthesis	Acc = 93.9%	GoogLeNet
Varcin, 2021 [52]	X-rays	2707	Vertebrae	Spondylolisthesis	Acc = 99.0%	Yolo v3 + MobileNet
Lee, 2019 [21]	Brain MRI and physiological	53	LBP	LBP diagnosis	Acc = 92.5%	SVM
Lamichhane, 2021 [22]	Brain MRI	51	LBP	LBP diagnosis	Acc = 78.7%	SVM
Lamichhane, 2021 [23]	Brain MRI	51	LBP	LBP diagnosis	Acc = 83.1%	Enet-subset + SVM
Shen, 2019 [24]	Brain MRI	90	LBP	LBP diagnosis	Acc = 79.3%	SVM
Mathew, 1988 [25]	Clinical data	200	LBP	LBP diagnosis	Acc = 82 to 90%	Inductive Learning
Staartjes, 2020 [26]	Clinical data	262	LBP	LBP diagnosis	Acc = 96.2%	Fuzzy rule-based classification on Chi’s method
Parsaeian, 2012 [27]	Clinical data	>34,000	LBP	LBP diagnosis	AUC = 0.75 and 0.75	Feedforward NN and Logistic Regression
Caza-Szoka, 2016 [53]	EMG signals	24	LBP	LBP diagnosis	Acc = 80%	Feedforward NN
Wang, 2019 [54]	EMG signals	288	LBP	LBP diagnosis	Acc = 92.9%	Spanning CNN
Liew, 2020 [55]	EMG and kinematic variables	49	LBP	LBP diagnosis	AUC = 0.97	Logistic Regression
Abdollahi, 2020 [56]	Kinematic variables	94	LBP	LBP diagnosis	Acc = 75%	SVM
Bishop, 1997 [57]	Kinematic variables	183	LBP	LBP diagnosis	Acc = 85%	Feedforward NN
Hu, 2018 [58]	Kinematic variables	44	LBP	LBP diagnosis	Acc = 97.2%	LSTM
Ashouri, 2017 [59]	Kinematic variables	53	LBP	LBP diagnosis	Acc = 96%	SVM
Karabulut, 2014 [60]	Biomechanical measures	310	LBP	LBP diagnosis	Acc = 89.7%	SMOTE, logistic model tree
Ketola, 2020 [61]	MRI	518	LBP	LBP diagnosis	Acc = 83%	Texture feature extraction and Logistic Regression
Torrado, 2021 [62]	PET imaging	33	LBP	LBP diagnosis	AUC = 0.88	Random Forest
Sanders, 2000 [63]	Pain drawings	250	LBP	LBP diagnosis	Sensitivity = 49%	Feedforward NN

Abbreviations: Magnetic Resonance Imaging (MRI), Electromyography (EMG), Positive Emission Tomography (PET), Low Back Pain (LBP), Accuracy (Acc), Area Under the Curve (AUC), Natural Language Processing (NLP), Convolutional Neural Network (CNN), Machine Learning (ML), Neural Network (NN), Support Vector Machine (SVM), Long Short-Term Memory (LSTM), Synthetic Minority Oversampling TEchnique (SMOTE).

Among the studies that aimed to diagnose LBP exploiting EMG signals and kinematic/biomechanical measures, Caza-Szoka et al. [53] performed a surrogate analysis of fractal dimensions from sEMG sensor array in order to identify a predictor of chronic LBP in 24 subjects, using a Feedforward NN with an accuracy of 80%. Wang et al. [54] proposed DeepLap, a system for the automatic diagnosis of LBP-symptomatic muscles. The system includes a belt for sEMG recording of lumbar muscles, and exploits a Spanning CNN for the recognition of symptomatic muscles; the model was validated on data of 288 patients with 92.9% accuracy. Liew et al. [55] used Logistic Regression on EMG signals and physiological parameters of 49 subjects for classifying LBP achieving an AUC of 0.97. Abdollahi et al. [56] used kinematic variables from a motion sensor to categorize 94 nonspecific LBP patients, using an SVM with an accuracy of 75%. Bishop et al. [57] used a Feedforward NN to classify 183 LBP patients using dynamic motion characteristics and achieving 85% accuracy. Hu et al. [58] used a Long Short-Term Memory (LSTM) NN on static-standing physiological variables of 44 subjects to diagnose LBP with an accuracy of 97.2%. Ashouri et al. [59] used an SVM to evaluate LBP from inertial sensor data of 53 subjects achieving an accuracy of 96%. Karabulut et al. [60] used Synthetic Minority Over-sampling TEchnique (SMOTE) preprocessing and Logistic Model Tree to predict LBP from biomechanical measures of 310 subjects with an accuracy of 89.7%.

Among the studies that aimed to diagnose LBP based on medical images related to the lumbar region, Ketola et al. [61] performed texture Feature Extraction and applied Logistic Regression on MRI images of 518 subjects to identify predictors of LBP, achieving an accuracy of 83%. Torrado-Carvajal et al. [62] used a Random Forest to state thalamic neuroinflammation as a discriminating signature for chronic LBP from Positive Emission Tomography (PET) images of 33 subjects, achieving an AUC of 0.88. Sanders et al. [63] used a Feedforward NN to develop an automated scoring of patients pain drawings of 250 subjects to identify LBP, achieving 49% sensitivity for a 5-class problem.

With regard to disc degeneration, the majority of the included studies used MRI imaging. Gao et al. [5] gave MRI images of 500 patients as an input to different CNNs, namely VGG-M, VGG-16, GoggleNet, and ResNet-34, in order to quantify disc degeneration, achieving a maximum accuracy of 86%. Ruiz-España et al. [29] extracted features from MRI images of 67 patients using Gradient Vector Flow, and tested several Machine Learning models to classify degenerated IVDs achieving accuracies greater than 90%. Oktay et al. [30] used MRI images of 102 patients as input for an SVM to classify degenerative disc diseases with an accuracy of 92.8%. Alomari et al. [31] used MRI images of 80 subjects to develop three Probabilistic Gaussian models related to disc appearance, location and context, in order to generate the inputs for a Gibbs probabilistic model to discriminate between healthy and unhealthy IVDs. Koh et al. [32] gave MRI images of 70 subjects as input to an ensemble of machine learning models composed of a perceptron classifier, a least mean square classifier, an SVM, and a k-Means, using a weighted sum of the models outputs in order to detect lumbar disc herniation, achieving 99% detection accuracy. Tsai et al. [33] trained a YOLO v3 CNN to detect lumbar disc herniation on MRI images of 168 subjects, achieving 81.1% accuracy after data augmentation. Pan et al. [34] used MRI images from 500 subjects to train a faster R-CNN to automatically diagnose disc bulging and herniation, with a mean accuracy of 88.8% over the five lumbar IVDs, after having performed IVDs localization and identification. Salehi et al. [37] used MRI images of 50 subjects to detect disc herniation using a K-nearest neighbor after having extracted features from the region of interest using Active Contour snakes and K-Means, achieving 97.9% accuracy. Beulah et al. [35] automatically segmented IVDs and extracted Gabor features from MRI images of 93 patients to discriminate between degenerated and healthy discs, achieving 92.5% accuracy using an SVM. Sundarsingh et al. [36] proposed Local Sub-Rhombus Binary Relation Pattern techniques to extract features from MRI images of 63 subjects to discriminate between healthy, bulging and desiccated discs. They achieved an average 94.7% accuracy feeding such features to a Random Forest classifier. Three additional studies diagnosed disc degeneration without using MRI imaging: Šušteršič et al. [38] used features extracted from force sensors embedded in a foot force platform in order to diagnose the type of disc herniation in 33 patients. They tested several machine learning models and achieved the best accuracy of 85% using a decision tree. Rankovic et al. [39] used measures extracted through the medium of a platform for the detection of foot pressure distribution in order to diagnose disc herniation on four different discs levels. They trained an adaptive network-based fuzzy inference system on data of 29 patients, correctly grading the side and level of herniation of 8 out of the 9 test subjects. Oyedotun et al. [40] used biomechanical measures of 310 subjects in the UCI Machine Learning Repository to train a feedforward NN to discriminate between healthy subjects and those suffering from disc herniation or spondylolistehesis. They achieved 92.5% accuracy on the three-class task, whereas they achieved 96.8% accuracy on the task of discriminating between healthy and unhealthy subjects.

With regard to the diagnosis of spinal stenosis, Han et al. [44] used a CNN named DMML-Net on MRI of 200 patients to diagnose lumbar neural foraminal stenosis with an average precision of 84.5%. Huber et al. [45] tested several machine learning algorithms for the lumbar spinal stenosis grading on 82 MRI, achieving 94% sensitivity and 98% specificity. Hallinan et al. [46] used a CNN to segment the spinal canal on MRI images of 446 patients, followed by a further CNN to detect different types of spinal stenosis, achieving accuracy scores of 96%, 92% and 89% for central canal stenosis, lateral recess, and neural foraminal stenosis, respectively. Won et al. [16] used a CNN to automatically grade spinal stenosis on MRI images of 542 patients achieving accuracy scores of 83.0% and 77.9% with respect to the ground truth evaluated by two different physicians.

With regard to the studies that addressed spondylolisthesis, Zhao et al. [50] used a Faster Adversrial Recognition Neural Network to detect vertebrae on MRI images of 150 patients, and used such detection system to grade spondylolisthesis, achieving 89.3% accuracy. Varcin et al. [51] used GoogLeNet, and compared its results to those achieved using AlexNet on X-ray images of 286 patients to diagnose the presence of spondylolisthesis, achieving 93.9% accuracy on images of 48 patients kept as the test set. In addition, the same group extended the study [52] by using a transfer learning-based CNN for spondylolisthesis detection; they extracted features from a total of 2707 images with a Yolo v3, and thus fed them to a fine-tuned MobileNet, achieving 99% test diagnosis accuracy.

However, some articles did not fall in any of the aforementioned categories. In the frame of routine clinical reporting, Lewandrowski et al. [28] used a Tiramisu NN and a CNN for reporting of 17,800 IVDs from MRI related to IVDs and spinal canal, achieving an accuracy of 85.2% for disc herniation. In the frame of scoliosis diagnosis, Adankon et al. [48] used 3D images of the surface of the human back of 165 patients, extracting features with local geometric descriptors, and feeding them to a least-squares SVM for the classification of scoliosis curve types, achieving 95% accuracy; Lin [49] fed X-ray images of 37 subjects to a Feedforward NN to diagnose scoliosis, with an identification rate of 84%. Veronezi et al. [47] used a Feedforward NN on X-ray images of 206 subjects to diagnose osteoarthritis, achieving an accuracy of 62.9%.

Finally, three articles aimed at the detection and classification of different lumbar structures and abnormalities at once: Jamaludin et al. [41,42] presented a CNN, namely SpineNet that achieved a detection accuracy of 71.5% for disc degeneration, 75.0% for disc narrowing, 95.2% for spondylolisthesis, 94.3% for stenosis, 86.3% for endplate defects, and 90.7% for marrow changes; Lehnen et al. [43] proposed a U-net for the identification of IVDs on MRI images of 146 subjects, and exploited measurement differences between the original and the segmented image for the detection of abnormalities, achieving an accuracy of 87% for disc herniation, 86% for disc extrusions, 76% for disc bulging, 98% for spinal canal stenosis, 91% for nerve root compression, and 87.6% for spondylolisthesis.

#### 3.1.2. Regression

Regression is a task that resorts to assign a numerical value to any input sample. Differently from classification, the number of classes is not predetermined; in other words, regression can be looked at as a classification task with an infinite number of classes. In this review, we identified a total of 12 papers performing a regression task as a CAD, and their main characteristics are reported in Table 3. In detail:

- 6 studies used MRI imaging;
- 4 studies utilized X-ray images (1 of which in combination with Moire images);
- 1 study employed CT images;
- 1 study exploited clinical data.

Vertebrae were the most investigated structures (5 papers), whereas other studies focused on IVDs, muscles, definition and quantification of LBP-related measures. In more detail, three studies focused on spinal deformity, three studies focused on the measurement of lumbar structures, two studies focused on the quantification of LBP, one investigated spondylolisthesis, and one assessed intramuscular fat quantification. It is worth noting that eight studies resorted to NNs and deep learning, two studies resorted to machine learning models, whereas two exploited threshold methods.

**Table 3:** Summary of the works performing regression.

Author/Year	Data Type	# Patients	Structures Involved	Task	Results	Model
Pang, 2019 [64]	MRI	215	30 lumbar spinal indices	Structure measurement	Total MAE = 1.22 mm	CARN
Neubert, 2014 [65]	MRI	7	Discs	Structure measurement	Errors: height = 4.1%, area = 0.1%	Active shape modeling
Niemeyer, 2021 [66]	MRI	1599	Discs	Pfirrmann grading	MAE = 0.08	CNN
Sneath, 2021 [67]	MRI	60	Discs	Disc ageing assessment	Age difference < 11 years	Ensemble of ML models
Natalia, 2020 [68]	MRI	515	Discs and spinal canal	Structure measurement	MAE = 0.9 mm	SegNet and Contour Evolution Algorithm
Sari, 2012 [69]	Clinical data	169	LBP	LBP quantification	Pain intensity error = 4%	Feedf. NN & Neuro-Fuzzy inference
Fortin, 2017 [70]	MRI	30	Muscles	Fat quantification	Reliability coefficient = 97–99%	Threshold
Chae, 2020 [71]	CT images	40	Vertebrae	Spinal deformity	Mean abs. Deviation = 1.4 to 3.5°	Decentralized CNN
Watanabe, 2019 [72]	Moire images + X-rays	1996	Vertebrae	Spinal deformity	Cobb angle MAE = 3.42°	CNN
Cho, 2020 [73]	X-rays	629	Vertebrae	Lordosis	MAE = 8.055°	U-net
Garcia-Cano, 2018 [74]	X-rays	150	Vertebrae	Spinal deformity	Cobb angle MAE = 4.79°	Ind. Comp. Analysis and Random Forest
Nguyen, 2021 [75]	X-rays	1000	Vertebrae	Spondylolisthesis	Mean deviation = 1.76°	CNN

Abbreviations: Cascade Amplifier Regression Network (CARN), Magnetic Resonance Imaging (MRI), Computed Tomography (CT), Low Back Pain (LBP), Mean Absolute Error (MAE), Neural Network (NN), Machine Learning (ML), Convolutional Neural Network (CNN).

With regard to papers focusing on spinal deformity, Chae et al. [71] developed a Decentralized CNN to evaluate spinal deformity on CT images of 40 subjects, achieving mean absolute deviation values ranging from 1.4 to 3.5°. Watanabe et al. [72] used a CNN to estimate spinal alignment on 1996 Moire images, with a Cobb angle MAE of 3.42°. Cho et al. [73] used a U-net for the automated Segmentation and measurement of lumbar lordosis on X-ray images of 629 patients, achieving an MAE on the curve angle of 8.06°. Garcia-Cano et al. [74] extracted features from X-ray images of 150 patients through the medium of Independent Component Analysis, and used Random Forest Regression to predict the spinal curve progression in adolescents with idiopathic scoliosis, achieving a Mean Absolute Error (MAE) of 4.79° for the Cobb angle.

With regard to the studies focusing on the measurement of lumbar structures, Pang et al. [64] used a Cascade Amplifier Regression Network (CARN) on MRI of 215 subjects for the quantification of 30 lumbar spinal indices, achieving an overall MAE of 1.22 mm. Neubert et al. [65] used Active Shape Modeling for the measurement of IVDs from MRI of seven patients, achieving estimate error of 4.1% and 0.1% for disc height and area, respectively. Natalia et al. [68] used a SegNet and a Contour Evolution Algorithm to measure anteroposterior diameter and foraminal widths in MRI images of 515 patients suffering from lumbar spinal stenosis with a mean error of 0.9 mm. Nguyen et al. [75] used a CNN trained on X-ray images of 1000 spondylolisthesis patients to measure structure deviation, achieving a mean deviation angle on 20 further test patients of 1.76°.

With regard to the studies focusing on LBP quantification, Sari et al. [69] tested a Feedforward NN and an Adaptive Neuro-Fuzzy inference system for the objective assessment of LBP intensity, using as input skin resistance and visual analog scale of 169 patients and achieving a pain intensity error of 4%.

In addition, Fortin et al. [70] used a threshold algorithm for Segmentation and quantification of paraspinal muscle composition with a reliability coefficient ranging between 97 and 99%. Niemeyer et al. [66] developed a CNN to frame the grading of the Pfirrmann as a regression problem, achieving an MAE of 0.08 on MRI images of 1599 subjects. Finally, Sneath et al. [67] proposed an ensemble of machine learning models to calculate a predicted “age estimate” for the age-related changes based on MRI images of 60 subjects, achieving a “predicted age” differing from the true subject age by less than 11 years in 80% of cases.

## 4. Discussion

The management of patients affected by spine-related problems, first LBP, is a demanding process which often involves gathering a thorough patient’s history, conducting a structured physical examination, and combining multiple imaging sources to accurately formulate the diagnosis and plan an appropriate treatment [76]. The use of multiple scales and measurement, as well as different imaging technologies, generates a vast amount of data which, while being fundamental to individualize the treatment approach, often becomes difficult to handle and fully interpret.

The advent of AI has been revolutionizing several research and clinical fields, including spine surgery, in which the development of automated systems may increase the accuracy and repeatability of the execution of tasks critical to the diagnostic process [2]. More specifically, the application of such tools—namely CAD systems—has been extensively reported in the recent literature with application to both conventional datasets (e.g., clinical data, lumbar MRI) and innovative technologies (e.g., brain fMRI, kinematic sensors). In this review, most included studies were focused on classification, through which AI systems are able to assign a numerical value to any input sample within a finite number of predetermined classes. Lumbar MRI was the main input source in the majority of studies. Indeed, investigated CAD systems were able to diagnose intervertebral disc degeneration based on IVD intensity at sagittal T2-weighted MRI images, with an accuracy of 86–92.8% [5,29,30,31]. In addition, several studies proposed different models for automatic classification of IVD degenerative changes based on the Pfirrmann grading system [5,29], while the preliminary manuscript from Oktay et al. [30] described a machine learning system able to discriminate between normal and degenerated IVDs only. Collectively, these studies showed an accuracy rate between 86% and 92.8%. Similarly, three studies showed a significantly high accuracy in detecting disc bulging and herniation, with rates of 81.1–99% [32,33,34]. Lewandrowski et al. [28] trained deep neural networks with a dataset of 17,800 IVDs and implemented it with a natural language processing (NLP) module capable of performing a sort of routine reporting for each disc level, achieving an accuracy of 81% for the diagnosis of foraminal stenosis, 86.2% for central stenosis, and 85.2% for disc herniation. In addition, other studies displayed CAD systems able to detect and rate central canal stenosis as well as foraminal and lateral recess stenosis, with an almost perfect or at least significantly high inter-reader agreement [44,45,46]. Jamaludin and colleagues developed a CNN capable of segmenting vertebrae and IVDs (with an accuracy of 95.6%) and to identify disc narrowing, marrow changes, endplate defects, spondylolisthesis, central canal stenosis as well as to perform Pfirrmann grading, with accuracy rates ranging from 70.1% to 95.4%. Furthermore, this model can directly mark disc and vertebral abnormalities in the form of heatmaps, namely “evidence hotspots” [41,42]. Similarly, Lehnen et al. [43] showed a CNN trained to segment the IVDs and detect disc herniation, extrusion, bulging, spinal canal stenosis, nerve root compression, and spondylolisthesis, with accuracy scores between 76 and 100%. In a study from Ketola and colleagues [61], a machine learning system showed accuracy, specificity, and sensitivity scores >80% in classifying patients as either symptomatic or nonsymptomatic based on LBP-related degenerative changes. However, the high incidence of false positives (asymptomatic individuals with disc degenerative changes) significantly impacted on the precision performance of the system.

X-rays were utilized as an input source only in two studies [47,49]. Veronezi et al. reported a significantly lower accuracy (62.85%) in recognizing osteoarthritic changes of the lumbar spine compared to other studies, due both to the heterogeneity of digital images and the low number of images used for training the system [47]. In another study, lumbar X-rays of scoliotic patients were utilized to build a 3D spine model and a multilayer feed-forward, back-propagation (MLFF/BP) Artificial NN was developed to identify the pattern of the scoliotic deformity [49]. However, AI applications for CAD are not limited to radiological images of the spine. Indeed, Lee et al. [21] have developed a system able to predict the intensity of LBP based on the integration of brain fMRI data and heart rate variability. The model demonstrated to anticipate the exacerbation of LBP in patients showing an increase of cerebral blood flow in the thalamus, prefrontal and posterior cingulate cortices and an increment of heart rate variability with an accuracy of 92.5%. In a similar study, Lamichhane and colleagues [22] showed that a machine learning approach was able to associate the reduction of cortical thickness in specific areas of the brain deputed to the elaboration of pain, emotions and vision in patients affected by LBP with an accuracy of 74.51%. In a subsequent analysis, the same authors tested a new hybrid feature selection technique (namely Enet-subset) to extract local graph measures from functional connectomes and determine their capacity to predict LBP using an SVM, achieving an average classification accuracy of 83.1% [23]. The alteration of visual network connectivity in individuals with chronic LBP was also documented by Shen et al. [24], who reported an accuracy rate of 79.3% in distinguishing patients with LBP in their machine learning study. On the other hand, Torrado-Carvajal and coauthors demonstrated the accumulation of the glial activation marker 18 kDa translocator protein (TSPO) in the thalamus of patients with chronic LBP using PET imaging and a Random Forest system [62].

The use of AI has been exploited in the diagnosis of LBP from clinical data as well. A preliminary study from Mathew et al. [25] showed that AI was able to outperform clinicians in the differential diagnosis of LBP, sciatica, or other spinal pathology already in 1988. Other studies have demonstrated the possibility of training AI systems to anticipate the diagnosis of lumbar disc herniation, lumbar spinal stenosis and chronic LBP based on patients’ performances during the five-repetition sit-to-stand test [26], predict the risk factors associated with LBP from a population survey [27], refine the diagnosis and personalize the treatment of LBP in a primary care context using free-text clinical notes [77] and automatically score pain drawings [63]. Additional inputs utilized to develop CAD systems for LBP diagnosis include sEMG during weightlifting [55] or an endurance test [53], as well as spinopelvic parameters [40,60] and kinematic data during static standing [58], trunk flexion/extension and lateral bending [56,57,59], which were able to detect LBP in affected patients with an accuracy >80%. Šušteršič et al. [38] tested five different classifier algorithms to diagnose the side and level of disc herniation based on the force exerted during normal standing or leaning either towards the forefeet or the heels. Using a Random Forest algorithm, the system reached an accuracy of 87.9%. Adankon and colleagues [48] proposed an SVM able to classify a scoliotic deformity based on a 3D model of patients’ spines built with four optical digitizers, reaching an overall accuracy of 95%.

Several studies have described the use of CAD systems for regression tasks, such as calculation of radiological indexes and LBP quantification. The investigations from the groups of Pang [64] and Neubert [65] presented automated systems able to extract numerous quantitative measurements from lumbar spine MRI, including vertebral height as well as disc height and area, whereas Natalia et al. [68] reported a model capable of calculating foraminal width and canal diameter following automatic segmentation of the surrounding structures. In each of these studies, the mean average error was not higher than 1.22 mm. Similarly, the system presented by Niemeyer et al. [66] showed to perform intervertebral disc degeneration grading with an average sensitivity >90%. An interesting study by Sneath et al. utilized a machine learning technique to gather degenerative changes of the spine and surrounding structures in order to perform an estimation of patients’ age, which eventually was within 11 years of the subjects’ physical age [67]. In addition, the AI systems proposed by Chae [71] and Cho [73] were able to automatically calculate several spinopelvic parameters predictive of lumbar spine deformity using lumbar X-rays, reaching an average error range of 1.45–3.51° in the former and 8.055° in the latter. With regard to scoliosis, Watanabe et al. [72] utilized a CNN able to estimate vertebral position, Cobb angle, and vertebral rotation using a combination of X-rays and Moirè topography, with a mean average error of 5.4 mm, 3.42° and 2.9°, respectively. In another study, 3D models of scoliotic spines were built from X-rays and updated every three months for 18 months to check for curve progression. Subsequently, a Random Forest system was trained with such a dataset and demonstrated to predict curve progression with a difference <5° compared to the real curvature [74]. Differently, Fortin and colleagues were the only ones to analyze paraspinal muscle composition in patients with LBP, reaching an intra-rater reliability coefficient of 0.95–0.99 [70]. Another study has described an AI-based model able to predict the severity of LBP based on skin resistance and pain expressed through visual analog scale (VAS) with an error of 4% [69].

Collectively, the majority of included studies showed a high degree of accuracy and accordance with conventional techniques while opening new perspectives in the diagnosis and treatment of LBP, as well as boosting time-consuming tasks and providing new insight from otherwise unused data. The identification and grading of lumbar degenerative changes remain the most investigated task with the highest performance rates compared to other studies [5,28,29,30,31,32,33,34,41,42,43,44,45,46,61,65,66,67,68,70]. Nonetheless, several studies have employed CAD systems to elaborate data from different sources, including functional imaging [22,23,24], biosensors [38,48,56,57,58,59], clinical data [38,48,56,57,58,59], etc., with significant results.

**Figure 4:**
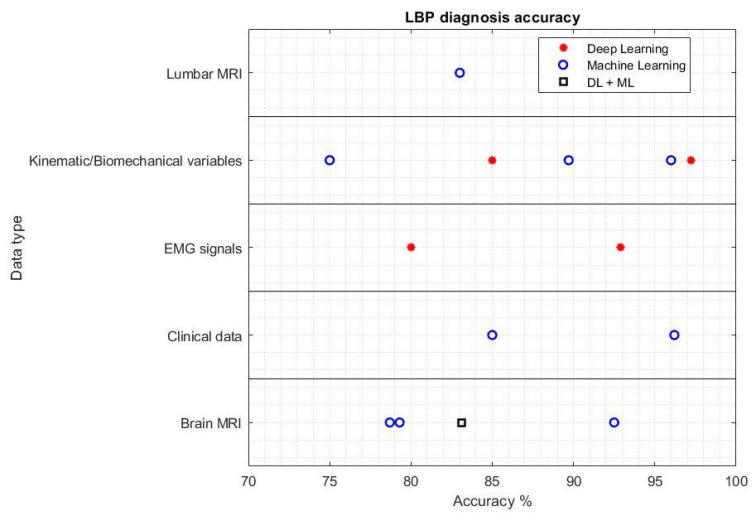
Accuracy of the LBP diagnosis task of studies using different features, reported on the vertical axis, and both deep learning (red asterisks), machine learning (blue circles) or both (black square) approaches.

**Figure 5:**
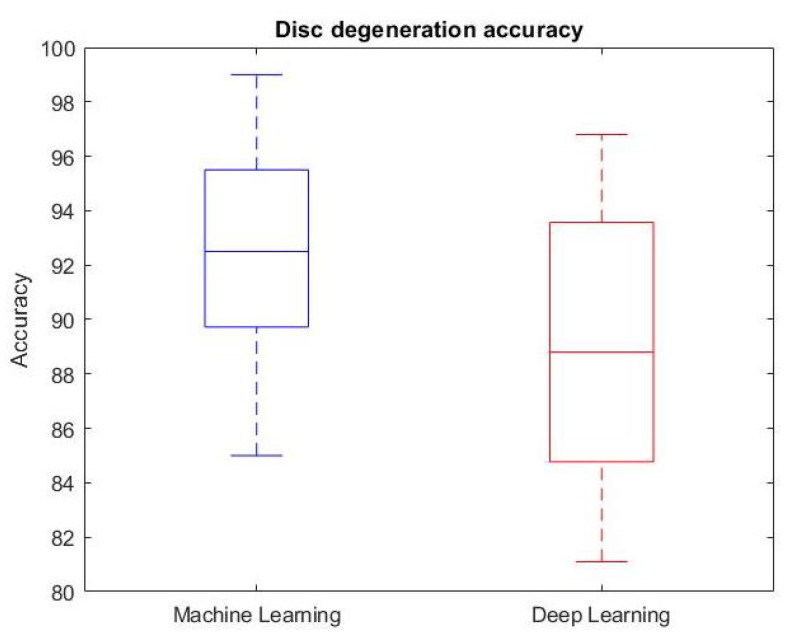
Boxplot reporting the accuracy of the disc degeneration classification task of studies that used machine learning (**left**) or deep learning (**right**) approaches.

With regard to the classification task, most studies addressed LBP diagnosis or disc degeneration. Figure 4 reports the accuracy of methods aiming at the diagnosis of LBP or, in other words, at the classification of whether or not a subject is suffering from LBP. The reported results differ on the type of data considered as model input, and on whether machine or deep learning techniques were utilized. The accuracy results are all greater than 75%, and three studies achieved accuracy greater than 95%, reaching a human-level diagnosis capability. Two of them exploited kinematic or biomechanical measures [58,59], whereas one exploited clinical data [26]. It is worth noting how the best performance was achieved by a deep LSTM net [58], although the majority of studies exploited machine learning techniques. Figure 5 presents a boxplot that reports the accuracy of the disc degeneration classification task. This boxplot considers nine studies that used machine learning (median accuracy = 92.5%), and four studies that used deep learning (median accuracy = 88.8%) techniques. Briefly, machine learning techniques achieved slightly better results, both in terms of median accuracy and best performance. However, it must be taken into account that the number of studies performing such a task was not sufficient to provide a thorough statistical analysis, and the same applies to the LBP diagnosis task. Thus, these results should be intended as a preliminary effort to identify the most promising approach in the frame of CAD applications to LBP. Finally, with regard to regression, there is no one task that is addressed more than the others, but rather each research group focused on a characteristic task. Nonetheless, some technically-sound studies have been presented, and their results are noteworthy when considering a specific task.

The implementation of AI systems in healthcare, particularly in terms of tools implying a direct clinical repercussion in the formulation of diagnosis or clinical decisions, is undoubtedly determining a paradigm shift, with significant ethics and regulatory issues [2]. More specifically, although apparently autonomous, such systems must be always accompanied by the judgement of the clinicians with regard to the diagnostic process. Furthermore, exceptional care should be taken considering the huge amount of personal data used to train AI systems in order to avoid the unintended divulgation of private information.

This study has some limitations. First of all, the significant heterogeneity across studies in terms of methodology, data source and outcomes prevented a meta-analysis to be performed. Second, as the search included English manuscripts only, we may have missed articles written in other languages matching with our inclusion criteria.

## 5. Conclusions

AI is undoubtedly revolutionizing medical research and patient care with its multiple applications in several fields, including spine surgery. In this study, we have systematically reviewed the available literature on the use of AI, and more specifically CAD, in supporting the diagnostic process in patients affected by LBP. The majority of included studies showed a high degree of accuracy and low margins of error in performing various tasks, most frequently identification of degenerative changes (disc degeneration or herniation, stenosis of the central canal and foramina, spondylolisthesis) while also presenting promising results from innovative data acquisition techniques. In this picture, the use of AI and CAD may effectively improve the diagnostic process and consequently patients’ outcomes.

## Figures and Tables

**Table 1:** Summary of the search words used on the PubMed research. The words in the medical or the AI group are connected by a logic OR, whereas the two groups of words are connected with a logic AND.

Medical Keywords		AI Keywords
Low Back Pain		
Lumbar		
Intervertebral disc degeneration		Artificial Intelligence
Intervertebral disc displacement		Machine Learning
Spine surgery	AND	Deep Learning
Spondylarthritis		Neural Network
Spondylarthrosis		Computer Aided Diagnosis
Spondylolisthesis		
Disc herniation		

## Data Availability

Not applicable.

## Supplementary Materials

The following supporting information can be downloaded at: https://www.mdpi.com/article/10.3390/ijerph19105971/s1, Table S1: Summary of the methodological quality of included studies regarding the four domains assessing the risk of bias of the QUADAS-2 score; Table S2: Summary of the methodological quality of included studies regarding the three domains assessing applicability concerns of the QUADAS-2 score.
