# Supplementary material for: Artificial Intelligence and Computer Aided Diagnosis in Chronic Low Back Pain: A Systematic Review
Source: Int J Environ Res Public Health. 2022 May 14;19(10):5971. doi: 10.3390/ijerph19105971 (PMC9141006; doi:10.3390/ijerph19105971)
Supplement: Supplementary file 1 [file ijerph-19-05971-s001.zip › ijerph-1659905-Supplementary_materials.pdf]

Table S1: Summary of the methodological quality of the included studies regarding the 4 domains (D1, D2, D3, and D4) assessing the risk of bias of the QUADAS-2 score

Study	D1	D2	D3	D4
Lewandrowski 2020	Unclear	Low	Low	Low
Gao 2020	Low	Low	Low	Low
Ruiz-Espana 2015	Low	Low	Low	Low
Oktay 2014	Unclear	Low	Low	Unclear
Alomari 2010	Unclear	Low	Low	Unclear
Koh 2012	Low	Low	Low	Unclear
Tsai 2021	Low	Low	Low	Low
Pan 2021	Unclear	Low	Low	Low
Sustersic 2020	High	Low	Low	Low
Oyedotun 2016	High	High	High	Unclear
Jamaludin 2017a	Unclear	Low	High	Low
Jamaludin 2017b	Low	Low	Low	High
Lehnen 2021	Low	Low	Low	Low
Han 2018	High	Low	High	High
Huber 2009	High	Low	Low	Low
Hallinan 2021	Unclear	Low	Low	Low
Veronezi 2011	Unclear	Low	Low	Low
Adankon 2012	Unclear	Low	Low	Low
Lin 2007	Unclear	Low	Low	Unclear
Lee 2019	Low	High	High	Low
Lamichhane 2021a	High	High	High	Low
Lamichhane 2021b	High	High	High	Low
Shen 2019	High	High	High	Low
Mathew 1998	Unclear	Low	Unclear	Low
Staartjes 2020	High	Low	Low	Low
Parsaeian 2012	Low	Low	High	Unclear
Caza-Szoka 2016	High	Low	High	Low
Liew 2020	High	Low	High	Low
Abdollahi 2020	High	Low	High	Low
Bishop 1997	Low	Low	High	Low
Hu 2018	High	Low	High	Low
Ashouri 2017	High	Low	High	Low
Karabulut 2014	High	Low	Unclear	Unclear
Ketola 2020	High	Low	Unclear	Low
Torrado-Carvajal 2021	High	Low	High	Low
Sanders 2000	Low	Low	High	Low

Table S1 (continuing)

Pang 2019	High	Low	Low	Low
Neubert 2014	High	Low	Low	Low
Niemeyer 2021	Low	Low	Low	Unclear
Sneath 2021	Low	Low	Low	Low
Natalia 2020	Low	Low	Low	Unclear
Sari 2012	High	Low	High	Low
Fortin 2017	Low	Low	Low	Low
Chae 2020	Low	Low	Low	Low
Watanabe 2019	Low	Low	Low	Low
Cho 2020	High	Low	Low	Low
Garcia-Cano 2018	High	Low	Low	High
Salehi 2019	Unclear	Low	Low	Unclear
Won 2020	Low	Low	Low	Low
Beulah 2021	Unclear	Low	Low	Unclear
Sundarsingh 2020	Unclear	Low	Low	Unclear
Rankovic 2015	Unclear	High	High	Unclear
Zhao 2019	High	Low	Low	High
Varcin 2019	Unclear	Low	Low	Unclear
Varcin 2021	Low	Low	Low	Low
Wang 2019	High	Low	Low	Low
Nguyen 2021	Low	Low	Low	Low

Table S2: Summary of the methodological quality of the included studies regarding the 3 domains (D1, D2, and D3) assessing the applicability concerns of the QUADAS-2 score

Study	D1	D2	D3
Lewandrowski 2020	Low	Low	Low
Gao, 2020	Low	Low	Low
Ruiz-Espana, 2015	Low	Low	Low
Oktay, 2014	Unclear	Low	Low
Alomari 2010	Unclear	Low	Low
Koh 2012	Low	Low	Low
Tsai 2021	High	Low	Low
Pan 2021	Unclear	Low	Low
Sustersic 2020	Low	Low	Low
Oyedotun 2016	Low	High	High
Jamaludin 2017a	Low	Low	Low
Jamaludin 2017b	Low	Low	Low
Lehnen 2021	Low	Low	Low
Han 2018	Unclear	Low	Low
Huber 2009	Low	Low	Low
Hallinan 2021	Unclear	Low	Low
Veronezi 2011	Unclear	Low	Low
Adankon 2012	Low	Low	Low
Lin 2007	High	Low	Low
Lee 2019	Low	Low	High
Lamichhane 2021a	Low	Low	High
Lamichhane 2021b	Low	Low	High
Shen 2019	Low	Low	High
Mathew 1998	Low	Low	Low
Staartjes 2020	Unclear	Low	Low
Parsaeian 2012	High	Low	High
Caza-Szoka 2016	Low	Low	High
Liew 2020	Low	Low	High
Abdollahi 2020	Low	Low	High
Bishop 1997	Low	Low	High
Hu 2018	Low	Low	High
Ashouri 2017	Low	Low	High
Karabulut 2014	Low	Low	Unclear
Ketola 2020	High	Low	Low
Torrado-Carvajal 2021	Low	Low	Low
Sanders 2000	Low	Low	High

Table S2 (continuing)

Pang 2019	Low	Low	Low
Neubert 2014	High	Low	Low
Niemeyer 2021	Low	Low	Low
Sneath 2021	High	Low	Low
Natalia 2020	Low	Low	Low
Sari 2012	Low	Low	High
Fortin 2017	Low	Low	Low
Chae 2020	Unclear	Low	Low
Watanabe 2019	High	Low	Low
Cho 2020	Low	Low	Low
Garcia-Cano 2018	High	Low	Low
Salehi 2019	Unclear	Low	Low
Won 2020	Unclear	Low	Low
Beulah 2021	Unclear	Low	Low
Sundarsingh 2020	Unclear	Low	Low
Rankovic 2015	Unclear	High	High
Zhao 2019	Unclear	Low	Low
Varcin 2019	Unclear	Low	Low
Varcin 2021	Low	Low	Low
Wang 2019	Low	Low	Low
Nguyen 2021	Low	Low	Low
